# Stimulus Control Over Action for Food in Obese versus Healthy-weight Individuals

**DOI:** 10.3389/fpsyg.2017.00580

**Published:** 2017-04-13

**Authors:** Poppy Watson, Reinout W. Wiers, Bernhard Hommel, Victor E. A. Gerdes, Sanne de Wit

**Affiliations:** ^1^ADAPT Lab, Department of Developmental Psychology, University of AmsterdamAmsterdam, Netherlands; ^2^Amsterdam Brain and Cognition, University of AmsterdamAmsterdam, Netherlands; ^3^Habit Lab, Department of Clinical Psychology, University of AmsterdamAmsterdam, Netherlands; ^4^Cognitive Psychology Unit, Leiden UniversityLeiden, Netherlands; ^5^Leiden Institute for Brain and CognitionLeiden, Netherlands; ^6^Department of Internal Medicine, MC SlotervaartAmsterdam, Netherlands; ^7^Department of Vascular Medicine, Academic Medical CenterAmsterdam, Netherlands

**Keywords:** Pavlovian-to-instrumental transfer, obesity, habit, associative learning

## Abstract

In the current study we examined an associative learning mechanism by which food cues (signaling low- versus high-calorie food) can bias instrumental responses directed toward those foods. To investigate the clinical relevance of this mechanism, we used a computerized Pavlovian-to-instrumental transfer task and compared performance of 19 severely obese individuals to that of 19 healthy-weight controls matched for age, education and gender. During the response-priming test we exposed participants to both food pictures and to Pavlovian cues predictive of those food pictures, and examined their biasing effect on instrumental choice. As expected, obese participants showed higher priming rates for palatable, high-calorie foods (potato chips and chocolate) relative to low-calorie foods (lettuce and courgette) whereas healthy-weight individuals did not show a difference between priming rates for these two food types. We also included various measures of impulsivity as well as a slips-of-action task designed to investigate the balance between goal-directed and habitual behavioral control in these two groups. We did not find any evidence of increased impulsivity or reliance on a habitual strategy during the slips-of-action task, in obese participants.

**General Scientific Summary:** Our environment is full of cues signaling the availability of tasty, but often unhealthy, foods. This study suggests that severely obese individuals are particularly sensitive to high-calorie food cues whereas low-calorie food cues have little effect on their behavior.

## Introduction

It has been argued that maladaptive food seeking and excess weight gain can be best understood (and treated) from a learning theory perspective (Jansen, 1998; Bouton, 2011; Boutelle and Bouton, 2015). Using various associative-learning paradigms it has been demonstrated in carefully controlled laboratory settings that responding for food can be triggered by exposure to those food rewards either directly or indirectly (via Pavlovian cues previously associated with those food rewards; Watson et al., 2014, 2016). These effects are relevant for understanding the mechanism by which our obesogenic environment, filled with cues signaling the availability of tasty food, can lead to maladaptive food-seeking behavior and ultimately to obesity (Cohen, 2008; Swinburn et al., 2011; Johnson, 2013). Therefore, the current study aimed to investigate the clinical relevance of this associative mechanism by comparing the degree to which food-associated pictures biased instrumental responding for those pictures in a group of severely obese individuals relative to healthy-weight controls.

**FIGURE 1:**
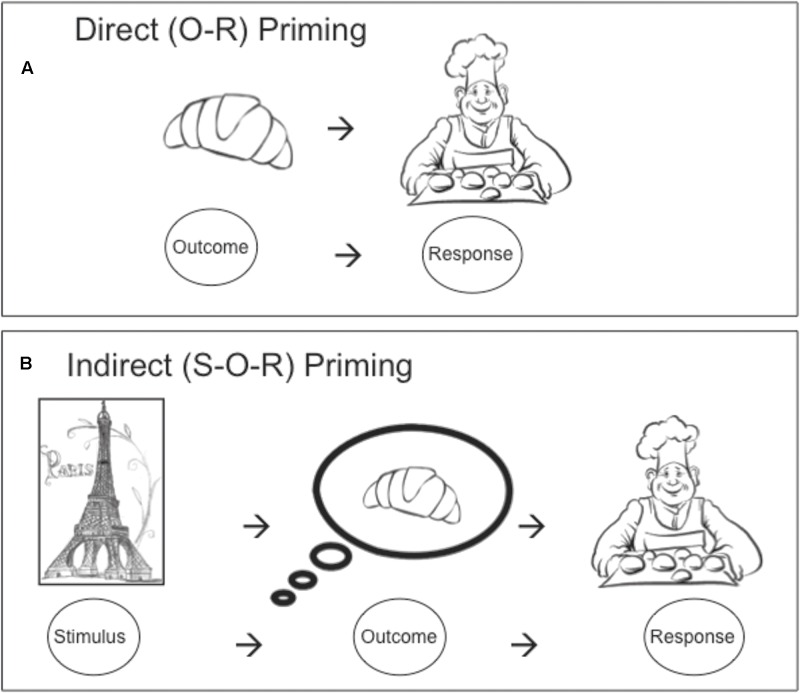
**(A)** Direct response priming – the sight of a croissant triggers a trip to the bakery. **(B)** Indirect response priming – seeing a painting of Paris reminds one of croissants, triggering a trip to the bakery.

The smell of a freshly baked croissant can trigger the action of visiting a bakery. This direct outcome-response (O-R) priming effect (see **Figure 1**) has been observed in the lab with demonstrations that presentation of pictures of, e.g., chocolate on a computer screen can elicit key presses that previously yielded a chocolate reward (Hogarth and Chase, 2011; Hogarth, 2012; Watson et al., 2016). However, even merely being reminded of croissants (e.g., by seeing a painting of Paris) can trigger the trip to the bakery (see **Figure 1**). This indirect stimulus-outcome-response (S-O-R) priming effect (with Pavlovian stimuli that had been paired with food outcomes) has been demonstrated experimentally with the Pavlovian-to-instrumental (PIT) task (Bray et al., 2008; Prévost et al., 2012; Lovibond and Colagiuri, 2013; Watson et al., 2014, 2016). Watson et al. (2014), for example, presented participants with abstract (Pavlovian) pictures that had previously been associated with popcorn or chocolate Smarties, while participants were free to respond for these two food rewards. The abstract pictures biased responding for the signaled reward, meaning that participants responded more for popcorn in the presence of the Pavlovian popcorn-associated picture (and likewise for Smarties). Crucially, these abstract pictures had never been directly paired with a response and could, therefore, trigger responses indirectly, via the expectancy of the food outcome; or in other words, via S-O-R associations. It has previously been argued that these associative response-priming mechanisms enable the obesogenic environment to trigger maladaptive food-seeking behavior (Swinburn et al., 2011; Watson et al., 2014, 2016). In support of this claim, we showed that the PIT effect is not diminished by specific satiation on the signaled food outcome (Watson et al., 2014) and that PIT in adolescents tends to be more pronounced with high- than with low-calorie snacks (Watson et al., 2016). To investigate this claim more directly, the present study investigates whether S-O-R priming with high-calorie snacks is particularly potent in obese (as opposed to healthy weight) individuals.

**FIGURE 2:**
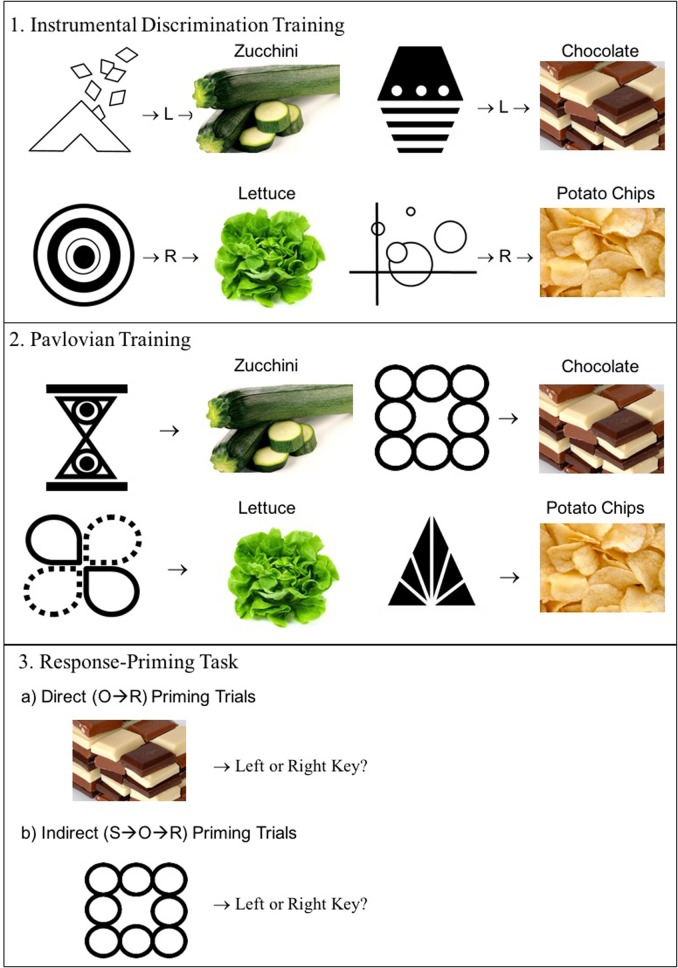
**Response-priming task design.** Participants first learnt the relationship between instrumental responses and food pictures (courgette, lettuce, chocolate, and potato chips). They then learnt the relationships between four Pavlovian logos and these same food pictures. Direct response priming occurs if presentation of the food pictures during the test phase triggers the previously associated response (e.g., chocolate → left key). Indirect response priming is observed if the Pavlovian logo triggers the response associated with the signaled food picture (e.g., Square logo → chocolate → left key).

We used an associative learning task (Watson et al., 2016) to investigate whether choice behavior of severely obese individuals is particularly vulnerable to the effect of external reminders of palatable, high-calorie food rewards (chocolate and potato chips) relative to low-calorie foods (lettuce and courgette). We compared high- and low-calorie food outcomes because unhealthy food choices are thought to be an important contributor to obesity (World Health Organization, 2015). Whilst chocolate and potato chips differ in flavor profile they are both palatable and high in calories. Courgette and lettuce were chosen as the low-calorie food outcomes because although they have a similar flavor profile, they are matched in palatability and calorie content (unlike for example fruit which tends to be high in sugar and therefore more palatable). During an initial instrumental training phase, participants were instructed to earn food pictures (and points) by pushing specific keyboard keys. Discriminative stimuli (abstract logos) signaled which key press would be rewarded on each trial. For example, in the presence of one logo a left key press led to a picture of chocolate and another logo signaled that a right key press led to a picture of lettuce (**Figure 2**, top panel). This training should lead to the formation of O-R associations. Both response keys were paired with one high- and one low-calorie food picture so as to prevent the development of a response bias. Subsequently, during Pavlovian training, participants learned the S-O relationships between different Pavlovian logos and these same food pictures (**Figure 2**, bottom panel). Finally, in the critical response-priming test, participants were shown a series of Pavlovian logos and food pictures, and were instructed to quickly and spontaneously press a key when they saw one of the images appear on the screen. We measured the extent to which instrumental responses would be primed both directly (by the food pictures; O-R) and indirectly (by the Pavlovian logos previously associated with the food pictures; S-O-R). Given findings that obese individuals find high-calorie foods more rewarding than low-calorie foods (Mela, 2006; Stoeckel et al., 2008) we expected that they would form stronger associations between Pavlovian cues, responses and high-calorie foods relative to low-calorie foods, during the training phases. During the response-priming test, high-calorie food pictures would then more readily prime the associated instrumental response. By contrast, we expected healthy-weight participants to show less of a differential response-priming effect between high and low-calorie outcomes.

**FIGURE 3:**
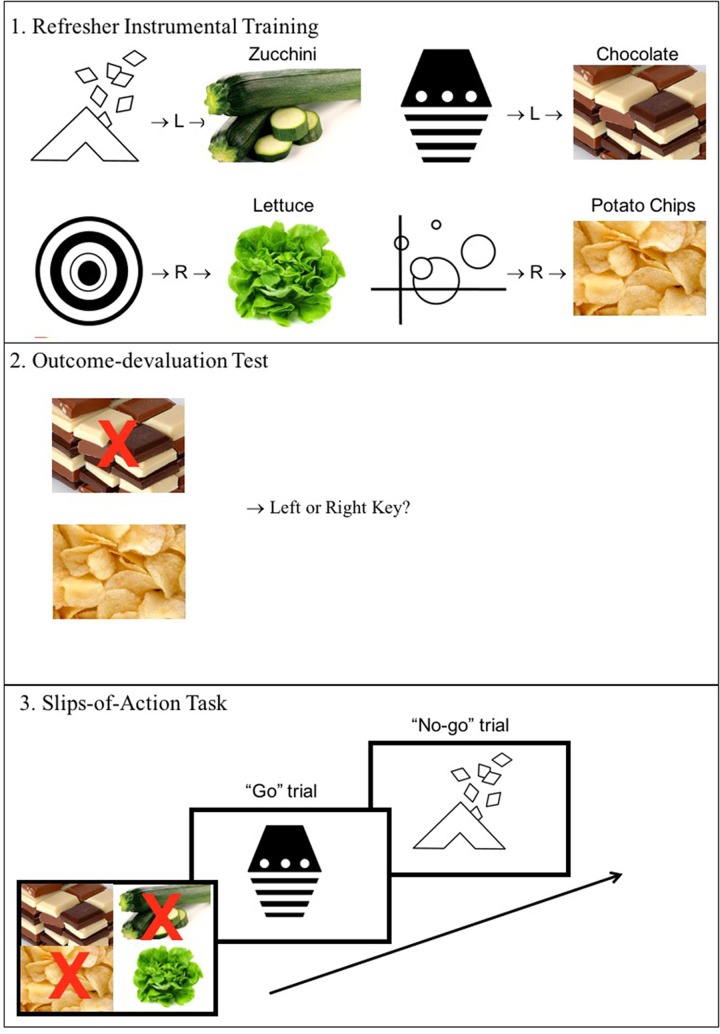
**Slips-of-action task design.** Participants first repeated the instrumental training phase. In the instructed outcome-devaluation test they then had to select the key that led to the still-valuable outcome (no red cross). Finally they had to memorize which food outcomes were still valuable and then decide upon presentation of every logo whether or not they should respond for the outcome it signaled.

Food-related cues can trigger responses via expectancy of the food outcome (as outlined above), but can also trigger over-learned responses via a habitual S-R mechanism. Under this habitual account, S-R associations gradually build up over a discriminative training phase such that eventually presentation of the discriminative stimuli can trigger the previously learned response directly, even when the resulting outcome is no longer valuable (a “slip of action,” see: de Wit and Dickinson, 2009). Slips of action occur in everyday life – for example when you decide that you are fully satisfied after the main course of dinner and don’t need dessert, but then mindlessly reach for a sweet anyway. To investigate whether obese individuals would be more prone to relying on S-R habits and make more ‘slips of action’ for outcomes that are no longer valuable, we also included a slips-of-action test in the present study (Gillan et al., 2011; de Wit et al., 2012; Dietrich et al., 2016). During this task, participants were asked in the presence of discriminative stimuli, to flexibly inhibit previously learned instrumental responses when the outcomes were no longer valuable (see **Figure 3**). A number of instrumental learning paradigms have previously been used to show that patients with obsessive-compulsive disorder, alcohol addiction, binge-eating disorder (BED) and obesity are generally less flexible in their behavior (Coppin et al., 2014; Zhang et al., 2014) and more prone to relying on habits (Gillan et al., 2011; Sjoerds et al., 2013; Horstmann et al., 2015; Voon et al., 2015). However, surprisingly, one recent study using the slips-of-action task did not find evidence for increased reliance on S-R habits in an obese sample (Dietrich et al., 2016). We therefore included this task in order to investigate this issue further.

Finally, to investigate the relationship between impulsivity, obesity and cue-elicited response priming, we included a self-report measure (the Barratt’s Impulsivity Scale, BIS-11; Patton et al., 1995) and a Stop Signal Reaction Time task (SSRT; Logan et al., 1997). We expected obese individuals to score higher on our measures of impulsivity compared to the healthy-weight individuals – in line with previous literature reporting higher scores for obese individuals on both self-report measures (Terracciano et al., 2009; Mobbs et al., 2010) and response inhibition tasks (Nederkoorn et al., 2006a,b; see review: Schag et al., 2013). Furthermore, individuals with high trait impulsivity are argued to be more sensitive to reward-associated cues (Carver and White, 1994; Stanford et al., 2009; Muhle-Karbe and Krebs, 2012). We expected, therefore, to find a positive correlation between impulsivity and response-priming rates for high-calorie foods (within each group).

In summary, the aims of the current study were to assess whether choice behavior of obese individuals is more sensitive to the biasing effect of food-associated cues, particularly in the context of palatable, high-calorie food. Both direct (O-R) and indirect (S-O-R) forms of response priming were examined and were related to individual differences in performance on the slips-of-action task and impulsivity.

## Materials and Methods

### Participants

Following approval by the University of Amsterdam Psychology Ethics Committee, patients who were in the process of preparing for bariatric surgery to reduce their BMI were recruited from a local hospital (MC Slotervaart). Researchers approached individuals in the waiting room and asked if they would like to take part in a 90-min study investigating memory for which payment of 15 was offered. Pre-screening of participants was not possible. Twenty-eight obese individuals (BMI > 30) were subsequently tested and none of the invited patients had been assessed by the psychologist and dietician of the bariatric surgery team as meeting the DSM criteria for Binge Eating Disorder (BED). Following testing, four obese participants were excluded (one was post-operative, one was not a patient, one had a learning disability, one had short-term memory problems following an accident). Another five obese participants who reported having an axis 1 disorder (ADD, mood disorder, substance addiction) and/or who were taking psychoactive medication were excluded. The only exception to this was a diagnosis of depression and use of SSRIs (selective serotonin se-uptake inhibitors), which were considered to be acceptable for inclusion. After exclusions, 19 obese participants remained, of whom four had previously been diagnosed with depression and were taking SSRIs.

Concurrently, 23 healthy-weight participants were recruited via advertisements on the University of Amsterdam website (testuva.nl). Interested individuals were asked to complete demographic information including age, height, gender, education level, and weight. Those with a BMI in the healthy range (18–25) were then invited for testing. As it became apparent that this sample was not going to be well matched to the obese group (in regards to gender, age and education) it was necessary to recruit a further 25 individuals from the wider community (via word of mouth and advertisements on a number of websites: proefbunny.nl, digiprik.nl). Eight control-group participants had underestimated their weight and were subsequently excluded because their BMI was too high (>25 kg/m^2^). None of the remaining participants reported having an axis 1 disorder or medication use. Without reference to performance data, 19 control-group participants were selected from the remaining sample pool of 40 individuals, based on gender, age and education profiles (for completeness the analysis was repeated with all 40 healthy-weight participants, see results). Detailed demographic information can be found in **Table 1**.

**Table 1:** Demographics of the sample.

	Obese group	Control group	Group differences
Group size (*n*)	19	19	
Gender ratio M:F	2:17	3:16	
Age (*SD*)	43.9 years (10.6 years)	45.0 years (14.0 years)	*t*(36) = 0.3, *p* = 0.80
BMI (*SD*)	44.0 (7.1)	23.0 (1.6)	*t*(36) = 12.6, *p <* 0.0001, *d* = 4.1, 95% CI = [2.9, 5.2]
Education ratio	3:11:5	3:8:8	χ^2^ (2, *n* = 19) = 1.2, *p* = 0.56
high school: vocational college: university			n.b. some cells have less than five entries.
BIS total score (*SD*)	63 (9)	61 (12)	*t*(36) = 0.34, *p* = 0.74
SSRT (*SD*)	267 ms (70 ms)	268 ms (108 ms)	*t*(36) = 0.05, *p* = 0.96
DEBQ external eating (*SD*)	3.3 (0.6)	2.8 (0.6)	*t*(36) = 2.5, *p* = 0.02, *d* = 0.81, 95%CI = [0.14,1.5]
Pre-test hunger rating (*SD*)	26% (29%)	30% (29%)	*t*(36) = 0.4, *p* = 0.66
Pre-test desire for high calorie(*SD*)	47% (30%)	28% (25%)	*t*(36) = 2.1, *p* = 0.04 *d* = 0.68, 95% CI = [0.02, 1.3]
Pre-test desire of low calorie (*SD*)	28% (26%)	26% (21%)	*t*(36) = 0.3, *p* = 0.76
Pre-test stress rating (*SD*)	36% (25%)	20% (22%)	*t*(36) = 2.1, *p* = 0.04*, d* = 0.68, 95% CI = [0.02, 1.3]
Interim stress rating (SD)	30% (28%)	22% (22%)	*t*(36) = 1.1, *p* = 0.29*, d* = 0.34, 95%CI = [-0.30, 0.98]
Final stress rating (SD)	33% (32%)	16% (20%)	*t*(36) = 2.0, *p* = 0.06*, d* = 0.64, 95%CI = [-0.15, 1.3]


*BIS = Barratt impulsivity scale; SSRT = Stop signal reaction time; DEBQ = Dutch eating behavioral questionnaire*.

### Stimuli and Materials

#### Computerized Tasks

The response-priming task used was as outlined by Watson et al. (2016) – but with different images and cover story. The subsequent slips-of-action test phase was based on that of de Wit et al. (2012), and used the same pictures and responses as the response-priming task. The tasks are described in the Procedure (see also **Figures 2, 3**). Four black-and-white logos functioned as discriminative cues and another four logos functioned as Pavlovian cues (200 × 200 pixels). Photographs measuring 260 × 160 pixels of potato chips, chocolate, lettuce and courgette functioned as outcomes (see **Figure 2**).

#### Food Desire, Hunger, and Stress Questionnaire

Participants were asked to rate their hunger, stress, and desire for each food on 10-cm VAS scales marked with the anchors: “none,” “neutral,” and “very much.”

#### Questionnaires

Eating motivations were assessed with the external eating subscale of the Dutch Eating Behavioral Questionnaire (DEBQ: van Strien et al., 1986). The Barratt Impulsivity Scale (Patton et al., 1995) was used to measure impulsivity.

#### SSRT

Response inhibition was measured with the SSRT (Logan et al., 1997). Our version contained four blocks of 64 trials. A staircase-tracking procedure ensured that participants were able to inhibit on approximately 50% of trials. Following successful stopping the stop signal delay was increased by 50 ms, whereas following unsuccessful stopping the delay was decreased by 50 ms. Longer SSRTs indicate greater difficulty in inhibiting prepotent responses.

### Procedure

Participants were tested on a laptop. Obese participants were tested in a room at the hospital and control group participants were tested either at the University, or at their home (only if individuals could be tested alone, without distraction). Participants were first given a potato chip and small pieces of chocolate, lettuce and raw courgette. They then tasted each and completed the *food desire, hunger, and stress questionnaire*.

They were then given instructions for the *“Delicious Snack Game”* in which they were told as a cover story that they were driving along the motorway and they had to earn points by collecting as many items of food as possible from various food stores along the way. Two cinema passes for the three highest performers were offered as incentive. For all stages of the task described below, the experimenter showed the participants example trials from a booklet (with different logos and food pictures) and confirmed that the instructions were clear before continuing.

#### Instrumental Training Phase

The task began with the *instrumental discrimination training phase*. Different logos signaled to participants that a particular food was available and that a left or right key press was required to obtain it. Participants learnt by trial and error which key press would be rewarded in the presence of each of the four discriminative logos. Correct responses were followed by a picture of the food outcome, a cash register sound and one point was added to their total score (displayed on screen). Incorrect responses were followed by a buzzer sound and “0.” “Too late” was displayed if no response was recorded within 2 s. All feedback screens were displayed for 1 s. Across participants, each of the food pictures was paired with each of the Pavlovian logos (using permutation). For each participant, the two response keys were each paired with one high-calorie and one low-calorie food picture. Eighty trials contained ten blocks in which the four logos were randomly presented twice.

#### Pavlovian Training Phase

During the *Pavlovian training* phase, participants first passively viewed the screen and were asked to remember the relationships between four new logos and the same four foods (see **Figure 2**). Each trial started with a (Pavlovian) logo that was presented at the top of screen during 3 s, with one of the four food pictures appearing underneath the logo during the final second. The relationships between the logos and the food pictures were permutated across participants. After eight trials (two random presentations of the four logo-food combinations), participants were told that they would be tested on what they had just learnt. There then followed the active Pavlovian training phase. On each trial a logo was presented at the top of the screen and smaller versions of the four food pictures presented underneath in a 2 × 2 matrix. The position of each picture within the matrix was randomly determined. Participants had 3 s to click on the correct food image with the mouse. Feedback (1 s) was the full-size image of the correct food picture with either the number of points displayed above (“1” or “0” for correct or incorrect responses) or “too late” for response omissions. The active phase of Pavlovian training consisted of ten blocks in which the four logos were randomly presented twice (80 trials in total).

#### Response-Priming Test

On each trial of the *response-priming test* a logo or food picture was presented. Participants were instructed to select either the left or the right key as quickly as possible every time they saw a picture appear. If they weren’t sure which key to press, they were told to not think too hard about the correct response but to spontaneously select a key in a non-systematic order. They would not receive any feedback on their responses but they were told that they were still earning points. On each trial, one of the twelve pictures that had been used in the task was presented for 2 s or until a response was made. These twelve pictures comprised the four food pictures to assess direct priming, the four Pavlovian logo stimuli to assess indirect response priming and the four instrumental logo stimuli as a control condition. The response-priming test contained 2 blocks in which the 12 pictures were each presented twice in random order (48 trials in total). The ITI during the response-priming task varied between 1 and 2 s.

Participants were then offered a 5-min break before continuing with the slips-of-action test. They were first given a potato chip and small pieces of chocolate, lettuce and raw courgette. They tasted each and completed the *food desire, hunger, and stress questionnaire* for the second time (interim).

#### Refresher Instrumental Training

This phase began with four refresher blocks of *instrumental training* (32 trials) exactly as outlined above.

#### Outcome-devaluation Test

Participants were then tested on their knowledge of the O-R relationships during a brief *outcome-devaluation test* (de Wit et al., 2007). On each trial, two of the outcome pictures (either both low-calorie or both high-calorie) were presented for 2 s, one above the other (see **Figure 3**). One of the outcomes had a red cross superimposed to indicate that it was no longer worth any points. Participants had to press the key that had previously (during the instrumental training) led to the still-valuable food outcome. No feedback was given. During eight trials, each outcome was devalued (via instruction) four times.

#### Slips-of-action Test

Finally, participants performed the *slips-of-action* test. At the outset, participants were again instructed that they should not respond for devalued foods (those with a red cross through them). At the start of each of four blocks, the food outcomes were presented on screen, but two of these (one high and one low calorie) had a red cross through them to indicate that these would now lead to subtraction of points. Subsequently, a series of discriminative logos were each presented for 1 s. Participants were instructed to earn points by pressing the appropriate keys for logos associated with still-valuable outcomes (“go trials”) but to refrain from responding for logos associated with a now-devalued food item (“no-go” trials). The percentage of responses as a function of outcome value was measured and no feedback was given during the test. Each of the four logos was shown four times per block, and across eight blocks, each of the outcomes was devalued twice. We also administered a baseline version of the task that was identical except that participants now saw the four *logos* appear at the start of each block. Therefore, the stimuli were devalued instead of the outcomes. This baseline version was included to control for individual differences in working memory/response inhibition with the only difference being that, unlike the slips-of-action test, it does not require evaluation of an anticipated outcome. The order of the two tests was counterbalanced across participants.

Finally, participants completed the SSRT task, BIS, DEBQ and demographic questionnaires (including one final question on stress). Participants were weighed and their height measured by the experimenter and payment was given.

### Statistics

In order to investigate whether obese participants would form stronger associations between Pavlovian cues, responses and high-calorie foods relative to low-calorie foods in the training phases we used ANOVA to examine accuracy and RT (correct trials only) with the between-subjects variable group (obese/healthy weight) and within-subject variables block (1–10) and calorie content (high/low). To investigate whether obese individuals would show show stronger priming effects for cues predictive of high- versus low-calorie foods in the response-priming test (relative to healthy-weight controls) we used ANOVA to examine accuracy during the response-priming test with variables group, calorie and cue type (food picture/Pavlovian logo). Finally, to investigate whether obese individuals would make more slips of action for devalued outcomes, ANOVA was used to analyze the mean percentages of responding to logos associated with devalued outcomes (no-go trials) and still-valuable outcomes (go trials) as a function of task (baseline or slips-of-action test), calorie content and group. Greenhouse-Geisser *p* values are reported with the original degrees of freedom. A significance criterion of *p* = 0.05 was adopted and all reported *t*-tests were two-tailed.

## Results

### Participants

As can be seen in **Table 1**, the groups were matched on gender, age and education level. While there were no significant differences between the groups on pre-test hunger rating or desire for the low-calorie foods, the obese group did report higher levels of desire for the high-calorie foods. Contrary to expectations, the groups did not differ significantly on the BIS total score or SSRT. The obese group, however, scored significantly higher on DEBQ external eating and reported higher levels of stress at the beginning of the experiment (but not at subsequent measurements).

### Instrumental Training Phase

**FIGURE 4:**
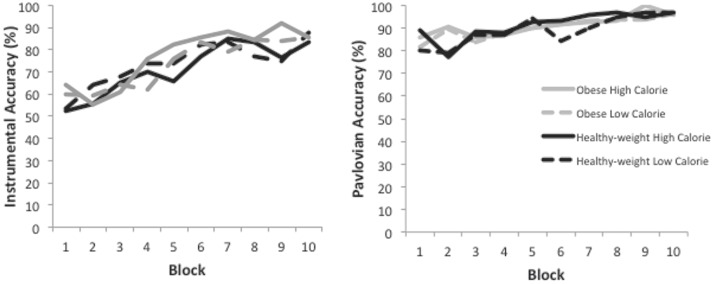
**(Left panel)** Accuracy over the 10 blocks of the Instrumental Training phase. **(Right panel)** Accuracy over the 10 blocks of the Pavlovian Training phase.

Participants had learned by the end of instrumental training which key press was required to successfully obtain food pictures with mean accuracy of 86% (*SD*: 20%) during the final block. Showing performance improvement over time, the accuracy analysis revealed a main effect of block, *F*(9,315) = 15.5, *p <* 0.0001, $\eta_{p}^{2}$ = 0.31, 95% CI [0.21, 0.36], (see **Figure 4**). There were no further significant results (all *ps* > 0.28). The RT analysis revealed a main effect of block only, *F*(9,171) = 4.8, *p* = 0.03, $\eta_{p}^{2}$ = 0.20, 95% CI [0.07, 0.27], indicating that participants became faster over the course of training. There were no further significant results (all *ps >* 0.27).

### Pavlovian Training Phase

The accuracy analysis confirmed that participants became more accurate over the course of Pavlovian training at associating particular logos with food outcomes, as indicated by a main effect of block, *F*(9,279) = 5.4, *p <* 0.0001, $\eta_{p}^{2}$ = 0.15, 95% CI [0.06, 0.20], (see **Figure 4**). There were no further significant results (all *ps >* 0.33). Likewise, for the RT analysis there was a main effect of block only, *F*(9,216) = 14.2, *p <* 0.0001, $\eta_{p}^{2}$ = 0.37, 95% CI [0.25, 0.44]. There were no further significant results (all *ps >* 0.14).

### Test Phase – Direct and Indirect Response Priming

**FIGURE 5:**
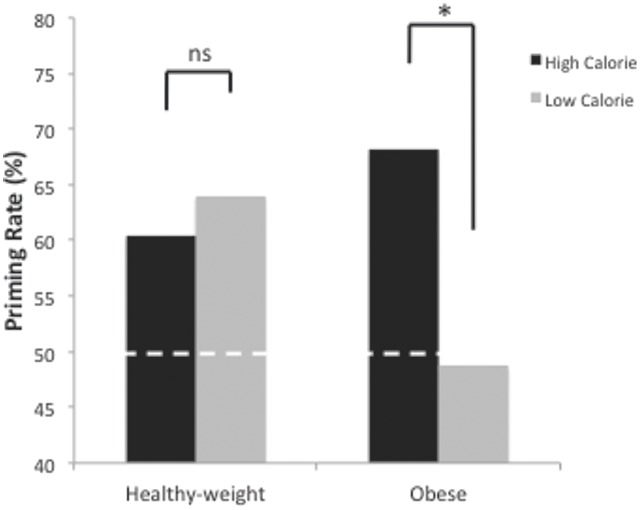
**Response-priming test.** Obese participants showed higher priming rates for high versus low-calorie outcomes. Performance did not differ for healthy-weight controls. White dotted line indicates 50% chance level. ^∗^*p* = 0.006.

Participants demonstrated that the discriminative associations from the instrumental training phase were still present with mean accuracy of 73% (*SD*: 23%) on trials in which the discriminative stimuli were presented. The data of interest were response-priming rates on trials where either the food pictures were presented (direct response priming) or the Pavlovian logos (indirect response priming). Trials were considered accurate (and priming successful) when participants selected the response that during instrumental training had yielded the outcome currently being presented/signaled. The mean priming rate was 60%, significantly higher than 50% chance level, *t*(37) = 3.7, *p* = 0.001, *d* = 0.59, 95% CI [0.24, 0.93]. The ANOVA analysis revealed an interaction between calorie and group, *F*(1,36) = 6.9, *p* = 0.01, $\eta_{p}^{2}$ = 0.16, 95% CI [0.008, 0.36], but there was no significant main effect of group (*p* = 0.52) nor any significant effects involving cue type (all *p*s > 0.17). The results are therefore shown collapsed across cue type in **Figure 5**. As can clearly be seen here, performance on high- and low-calorie trials did not differ significantly for participants in the healthy-weight group, *t*(18) = 0.58, *p* = 0.57, *d* = 0.18. In contrast, obese participants showed higher priming rates for the high-calorie versus the low-calorie outcomes, *t*(18) = 3.1, *p* = 0.006, *d* = 0.82. When comparing the two groups, the obese individuals did not differ from healthy-weight individuals in their priming rates for high-calorie food outcomes *t*(36) = 1.0, *p* = 0.32, *d* = 0.33, 95% CI [-0.31, 0.96], but did show significantly reduced priming rates for low-calorie food outcomes *t*(36) = 2.3, *p* = 0.03, *d* = 0.75, 95% CI [0.08, 1.4]. Finally, the RT analysis revealed no significant effects (all *p*s > 0.18).

### Refresher Instrumental Training

During the final block of the refresher training the mean accuracy was 90% for the obese group (*SD*: 17%) which did not differ significantly from the healthy-weight group mean accuracy 84% (*SD*: 22%), *t*(36) = 0.93, *p* = 0.36, *d* = 0.30, 95% CI [-0.34, 0.94].

### Outcome-devaluation Test

The ability of participants to select responses associated with still-valuable outcomes was examined during the outcome-devaluation test. The repeated measures ANOVA did not reveal any significant group or calorie effects for the accuracy measurement (all *ps >* 0.35), nor for the RT (all *ps >* 0.41).

### Slips of Action Task

One participant was excluded from the obese group as she did not understand the instructions and responded on all trials. For the remaining participants a significant interaction between task and value was observed, *F*(1,35) = 17.2, *p <* 0.0001, $\eta_{p}^{2}$ = 0.33, 95% CI [0.09, 0.52]. Further analysis revealed that participants performed better on the (simpler) baseline version of the task – responding successfully on 86% (*SD*: 17%) of go trials – significantly more than the slips-of-action test go-trial response rate of 72% (*SD*: 28%; *t*(36) = 3.7, *p* = 0.001, *d* = 0.56). Likewise, the no-go trials mean response rate was 16% (*SD*: 23%) during the baseline test, significantly less than responding for devalued outcomes during the slips-of-action test (mean: 25%, *SD*: 27%; *t*(35) = 2.3, *p* = 0.03, *d* = 0.34). There were no further significant effects (all *ps >* 0.13), meaning that the two groups did not differ significantly in their ability to selectively withhold responses for devalued (high-calorie) outcomes.

### Correlational Analyses

After confirming that the data were normally distributed we ran a number of correlational analyses (see **Table 2**). We calculated difference scores (high minus low calorie) for the mean response-priming rate and the desire ratings and correlated these variables across the entire sample. As can be seen in **Table 2**, this correlation was not significant suggesting that performance on the response-priming task does not simply reflect current desire for the different foods. In addition, no significant relationships were observed between the high-calorie response priming score and the DEBQ external eating score, BIS total impulsivity score, or SSRT performance. Interestingly, we did find that higher impulsivity (as reflected in poor SSRT performance) was related to poorer performance on both the slips-of-action task and the baseline test.

**Table 2:** Results from correlational analyses.

Difference (high minus low calorie) desire score × difference priming score	*r*(36) = 0.1, *p* = 0.40
High-calorie priming × desire score	Obese group*: r*(17) = -0. 01, *p* = 0.69 Control group: *r*(17) = 0.4, *p* = 0.08
High-calorie priming × DEBQ EE	Obese group*:*ρ(17) = -0.3, *p* = 0.16 Control group: ρ(17) = 0.2, *p* = 0.34
High-calorie priming × pre-test stress	Obese group*: r*(17) = -0.11, *p* = 0.65 Control group: *r*(17) = -0.17, *p* = 0.49
High-calorie priming × BIS total	*r*(36) = 0.06, *p* = 0.72
High-calorie priming × SSRT	*r*(36) = -0.1, *p* = 0.41
Slips of action (difference score valuable – devalued) × SSRT	ρ(35) = -0.4, *p* = 0.01
Slips of action (baseline test; difference score valuable – devalued) × SSRT	ρ(35) = -0.4, *p* = 0.03


*DEBQ EE = Dutch behavioral eating questionnaire external eating; BIS = Barratt impulsivity scale; SSRT = Stop signal reaction time*.

### Control Analyses

For the analyses presented thus far, we had excluded several healthy-weight participants in order to match the healthy-weight group with the obese group on age and education. For transparency sake, and also to boost the power of our analysis, we repeated all of the above analyses with inclusion of all 40 healthy-weight individuals. In this group (mean age 38.0 years, *SD*: 14.4 years), seven were male, and 70% (28) were university educated. Including these participants did not change the observed pattern of results and, crucially, during the response-priming task we replicated the Calorie × Group interaction *F*(1,57) = 13.1, *p* = 0.001, $\eta_{p}^{2}$ = 0.19, 95% CI [0.04, 0.35].

Given that serotonin levels are known to affect performance on the slips-of-action task (Worbe et al., 2015) we replicated the analyses from the slips-of-action task after exclusion of the four obese participants who had a diagnosis of depression and were using SSRIs. The significant interaction between task and value, *F*(1,31) = 16.2, *p* < 0.0001, $\eta_{p}^{2}$ = 0.34, 95% CI [0.09, 0.54], was replicated.

Finally, to justify inclusion of the four obese participants who had a diagnosis of depression and were using SSRIs we replicated the response-priming task analysis after exclusion of these four participants, again observing an interaction between Calorie × Group *F*(1,32) = 5.7, *p* = 0.023, $\eta_{p}^{2}$ = 0.15, 95% CI [0.00, 0.36].

## Discussion

This study demonstrates that for obese individuals the biasing effects of external food cues on instrumental choice behavior are particularly potent in the context of (palatable) high- relative to low-calorie food outcomes. When obese participants were directly shown pictures of potato chips or chocolate, or Pavlovian logos indirectly associated with these, they more frequently performed the response that had previously yielded that food outcome. By contrast, when presented with pictures of lettuce and courgette, or logos associated with these, obese participants pushed the two response keys equally often. Healthy-weight controls did not show this differential pattern of responding for high- versus low-calorie cues, and responded on the key associated with the presented/signaled picture regardless of calorie content. It has previously been suggested that these response-priming effects by external stimuli likely underlie maladaptive or addictive behavior (Hogarth and Chase, 2011; Watson et al., 2014; Colagiuri and Lovibond, 2015), and these results provide the first evidence of a maladaptive pattern of response priming by external stimuli in a clinically obese population. It should be noted that the obese group did not demonstrate stronger response priming for high-calorie cues *per se* (relative to the healthy-weight individuals) but that they showed markedly reduced responding for low-calorie cues. This suggests that whereas healthy weight individuals would be as likely to make a healthy food choice as an unhealthy one when strolling around in the cue-rich environment of the supermarket, obese individuals would be more biased by displays of high-calorie, tasty foods than for example, the vegetable section.

We found that logos previously associated with food pictures were able to trigger instrumental responses to the same degree as the food pictures themselves (see also Watson et al., 2016). The fact that food pictures and abstract stimuli predictive of these can bias choice reinforces the concerns that have been expressed about the obesogenic environment (Cohen, 2008; Harris et al., 2009; Swinburn et al., 2011), particularly as these response-priming effects are still observed when the food is currently undesired (e.g., after satiation; Watson et al., 2014). That the motivating properties of food can generalize to stimuli that have been associated with that food – and persist after satiation – underscores the powerful influence of an environment that constantly signals the availability of unhealthy, calorie-dense food, and the challenge that it poses for those who struggle to resist this temptation. From a clinical perspective it is unclear how to weaken learnt associations between cues, food and responses as these associations appear to be highly persistent and resistant to extinction (for reviews, see: Bouton, 2011; Boutelle and Bouton, 2015). Furthermore, a neuroimaging study with a similar paradigm provided evidence that this response priming effect is related to activity of the posterior putamen, a brain region that has previously been implicated in inflexible, habitual behavior (Bray et al., 2008). Nonetheless, bariatric surgery is arguably the most effective treatment for obesity (Gloy et al., 2013) and leads to reductions in self-reported craving and striatal neural responses to palatable food pictures (Ochner et al., 2011, 2012; Scholtz et al., 2013). It is possible, therefore, and an avenue for future research, that the group of obese individuals would post-operatively show a reduced response priming effect for high-calorie foods. It would also be interesting to examine a group of non-dieting obese in order to see whether obese individuals who do not have the goal of losing weight show stronger priming effects for high-calorie foods.

At first glance, the current results – and those of a previous study using the same design (Watson et al., 2016) – seem to contradict previous PIT studies that have demonstrated that these response-priming effects are *not* sensitive to changes in the motivational value of the food outcome. In contrast to our findings that response-priming effects are stronger in the context of more motivationally salient high-calorie food outcomes, many studies have demonstrated that food-associated cues will trigger responding for a snack even when participants have just been sated on that food (Rescorla, 1994; Holland, 2004; Corbit et al., 2007; Hogarth and Chase, 2011; Watson et al., 2014). Furthermore, related research with addictive substances has failed to relate response-priming effects to severity of substance dependence (Hogarth and Chase, 2011; Hogarth, 2012; Martinovic et al., 2014). To reconcile these contrasting findings, we argue that the motivational relevance of outcomes can modify the associative strength of S-O and O-R associations formed during training (Muhle-Karbe and Krebs, 2012; Watson et al., 2016) but that once the underlying associations have been established, this response-priming mechanism may not be flexibly modulated by changes in the motivational relevance of outcomes. Those aforementioned studies, for example, used foods that were equally appetitive during training and subsequently devalued one of the foods just before the response-priming test phase (Corbit et al., 2007; Hogarth and Chase, 2011; Watson et al., 2014). In contrast, in the current study participants already preferred the (tasty) high-calorie foods relative to the (bland) low-calorie foods at the start of the experiment. Further work is needed to investigate this hypothesis as different paradigms have been used in these two lines of research and the effect of pre- versus post-training motivational manipulations remains to be formally tested in the lab. An alternative explanation of the current results is that participants in the obese group were simply not motivated to perform on the low-calorie trials. Their performance during the training phases, however, argues against this interpretation, as accuracy and reaction times did not differ for high- versus low-calorie outcomes. Nor did we find evidence of a correlational relationship between desire ratings and response-priming rates.

Although we claim that the outcome-response associative mechanism can provide insight into exactly *how* the obesogenic environment can bias behavior, this is by no means intended to be a full account of the processes leading to the development and maintenance of obesity, which is a highly complex condition. We expected some of the personality and behavioral measures, such as impulsivity, to offer further insight but this was not the case. We did not find any evidence of increased impulsivity in the obese group relative to the healthy controls, which is at odds with reports that obese individuals are reportedly more impulsive (Nederkoorn et al., 2006a,b; Terracciano et al., 2009; Mobbs et al., 2010; Schag et al., 2013), particularly when responses are directed toward food pictures (Nederkoorn et al., 2012; Houben et al., 2014). In addition, in the current study, obese individuals did not make more slips of action in the presences of cues signaling devalued food outcomes. We failed, therefore, to provide evidence for increased habit propensity in obesity. This result is in line with that reported by Dietrich et al. (2016), but is nonetheless surprising given a previous finding that obese individuals are impaired at flexibly modulating responding on the basis of changes in outcome value (Horstmann et al., 2015). A difference between that study and the present one (together with the study by Dietrich et al., 2016) is, however, that they manipulated outcome value by satiating participants on the instrumental outcomes. With the latter design, it is possible that group differences in behavioral sensitivity to devaluation relate to differential efficacy of the satiation manipulation. Another interesting possibility to explore in future research is that obese individuals with BED may be more prone to form compulsive habits than the current sample (that did not show eating disorders). Task performance can markedly differ depending on the presence or absence of BED (Schag et al., 2013; Voon et al., 2015), with one study providing evidence that obese individuals with BED relied on inflexible, ‘model-free’ decision-making whereas those without performed as well as healthy-weight controls (Voon et al., 2015). To conclude, the precise personality traits that characterize a clinical group of severely obese individuals and their vulnerability to food-associated cues remain to be illuminated.

We should acknowledge the limitations of the current study. Unavoidably, the recruitment methods differed between the two groups which may have led to the patients reporting slightly higher levels of stress than the control participants at the beginning of the experiment (due to the hospital setting). However, the stress measurements between the two groups did not differ significantly when subsequently tested and reported stress at the beginning of the experiment did not correlate with priming rates for high-calorie food. In addition, it was necessary to exclude a number of obese individuals from the final data analysis which may have reduced power, particularly when examining the individual differences measures. Nonetheless, we had sufficient power to observe a marked difference between the groups in the degree to which food-related outcomes primed the previously learned instrumental response.

In summary, this study is the first to provide evidence for the clinical relevance of this associative response-priming mechanism by demonstrating a maladaptive pattern of responding in obese individuals in which high but not low-calorie food outcomes triggered responses directed toward those foods. This means that obese individuals may find it more difficult to make healthy food choices – an issue compounded by the fact that our obesogenic environment contains a plethora of cues signaling the availability of unhealthy, but rarely healthy, foods. Efforts should be made to prevent the formation of these learnt associations for unhealthy foods and further investigate ways to increase responses directed toward healthy foods.

## Ethics Statement

This study was approved by the UvA Psychology Ethics Committee. Participants were provided with an information brochure prior to participating. In this brochure, it was made clear to them that they could withdraw participation from the study at any point, and that their data would be fully anonymised. They then signed a consent form. All participants were of adult age. Some were in the healthy BMI range, and others in the obese BMI range.

## Author Contributions

All authors listed, have made substantial, direct and intellectual contribution to the work, and approved it for publication.

## Conflict of Interest Statement

The authors declare that the research was conducted in the absence of any commercial or financial relationships that could be construed as a potential conflict of interest.
